# Supplementary material for: Plasma Membrane Protein Nce102 Modulates Morphology and Function of the Yeast Vacuole
Source: Biomolecules. 2020 Oct 23;10(11):1476. doi: 10.3390/biom10111476 (PMC7690685; doi:10.3390/biom10111476)
Supplement: Supplementary file 1 [file biomolecules-10-01476-s001.pdf]

Supplementary materials

Table S1. Yeast strains used in the study.

Strain	Genotype	Source
BY4741	*MATa his3Δ1 leu2Δ0 met15Δ0 ura3Δ0*	*EUROSCARF*
BY4742	*MATα his3Δ1 leu2Δ0 lys2Δ0 ura3Δ0*	*EUROSCARF*
fhn1Δ	BY4742; *fhn1Δ::kanMX4*	*EUROSCARF*
nce102Δ	BY4742; *nce102Δ::kanMX4*	*EUROSCARF*
RH1800	*MATa his4 leu2 ura3 bar1–1*	H. Rietzman
Y001	BY4741; *nce102Δ*; *CAN1::GFP::LEU2* (YIp128)	Loibl et al., 2010
Y143	BY4742*; PMA1::GFP::LEU2* (YIp128)	Grossmann et al., 2007
Y193	BY4742; *SUR7::GFP::URA3* (YIp211)	Grossmann *et al.*, 2007
Y225	BY4742*; HXT1::GFP::LEU2* (YIp128)	This study
Y240	BY4742; *NCE102::GFP::URA3* (YIp211)	This study
Y249	BY4742; *PIL1::GFP::URA3* (YIp211)	This study
Y815 (*nce102Δfhn1Δ*)	*Sporulation, MATα, lys- met- leu- ura- his-*	This study
Y881	Y240; *NVJ1::mRFP::LEU2* (YIp128)	This study
Y970	*nce102Δ; SUR7-NCE102::GFP::URA3*	This study
Y1009	BY4742; *vps4::kanMX4; NCE102::GFP::URA3* (YIp211)	This study
Y1030	BY4741; *nce102Δ*; *CAN1::GFP::LEU2 (YIp128); SUR7-NCE102::mRFP::URA3*	This study
Y1031	BY4741; *CAN1::GFP::LEU2* (YIp128); *NCE102::mRFP::URA3* (YIp211)	Grossmann *et al.*, 2008
Y1039	BY4742; *TRP1::ss-dsRed-HDEL::URA3* (YIp211)*; NCE102::GFP::LEU2* (YIp128)	This study
Y1177	Y240; *VPH1::mCherry::clonNAT* (pFA6a-mCherry)	This study
Y1247	*nce102Δ*; *VPH1::GFP::LEU2* (YIp128)	This study
Y1248	Y815; *VPH1::GFP::LEU2* (YIp128)	This study
Y1249	*fhn1Δ*; *VPH1::GFP::LEU2* (YIp128)	This study
Y1250	BY4742; *VPH1::GFP::LEU2* (YIp128)	This study
Y1263	Y1247; *SUR7-NCE102::mRFP::URA3*	This study

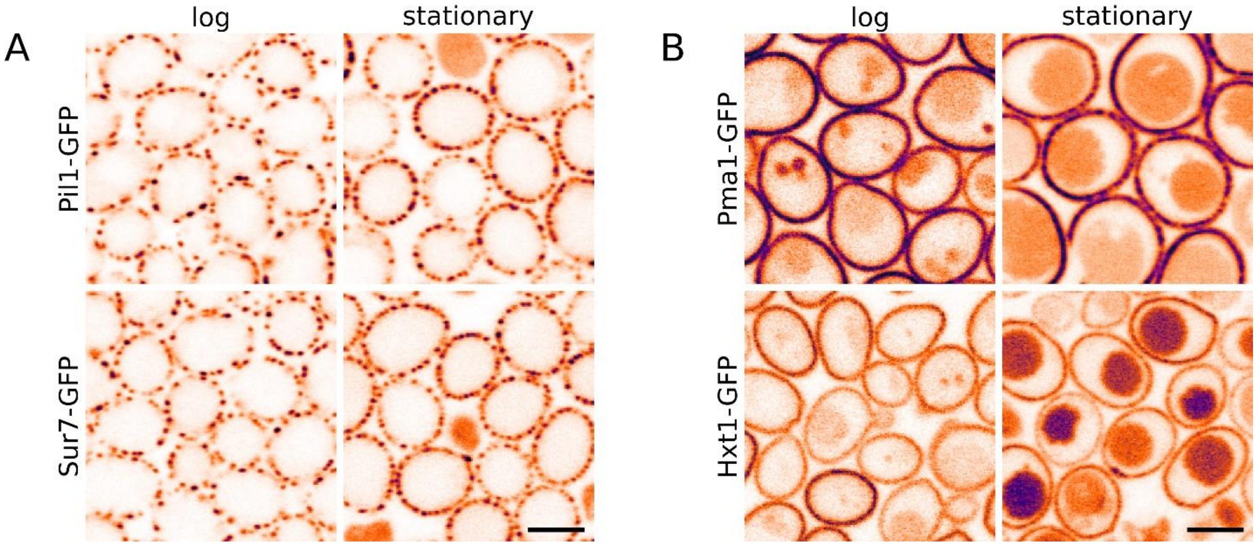

Figure S1. Changes in distribution of various plasma membrane proteins during gradual glucose depletion. Eisosome marker, Pil1, MCC marker, Sur7, the main proton ATPase, Pma1, and one of hexose permeases, Hxt1, were tagged with GFP (strains Y249, Y193, Y143, Y225, respectively) and localized in

cultures 6 h (log) and 48 h (stationary) after inoculation. Note the absence of MCC/eisosome proteins internalization in A, and fluorescence signal localized to vacuolar lumen (but not the vacuolar membrane) indicating the protein degradation in B. Transversal confocal sections are shown. Bars: 5 μm.

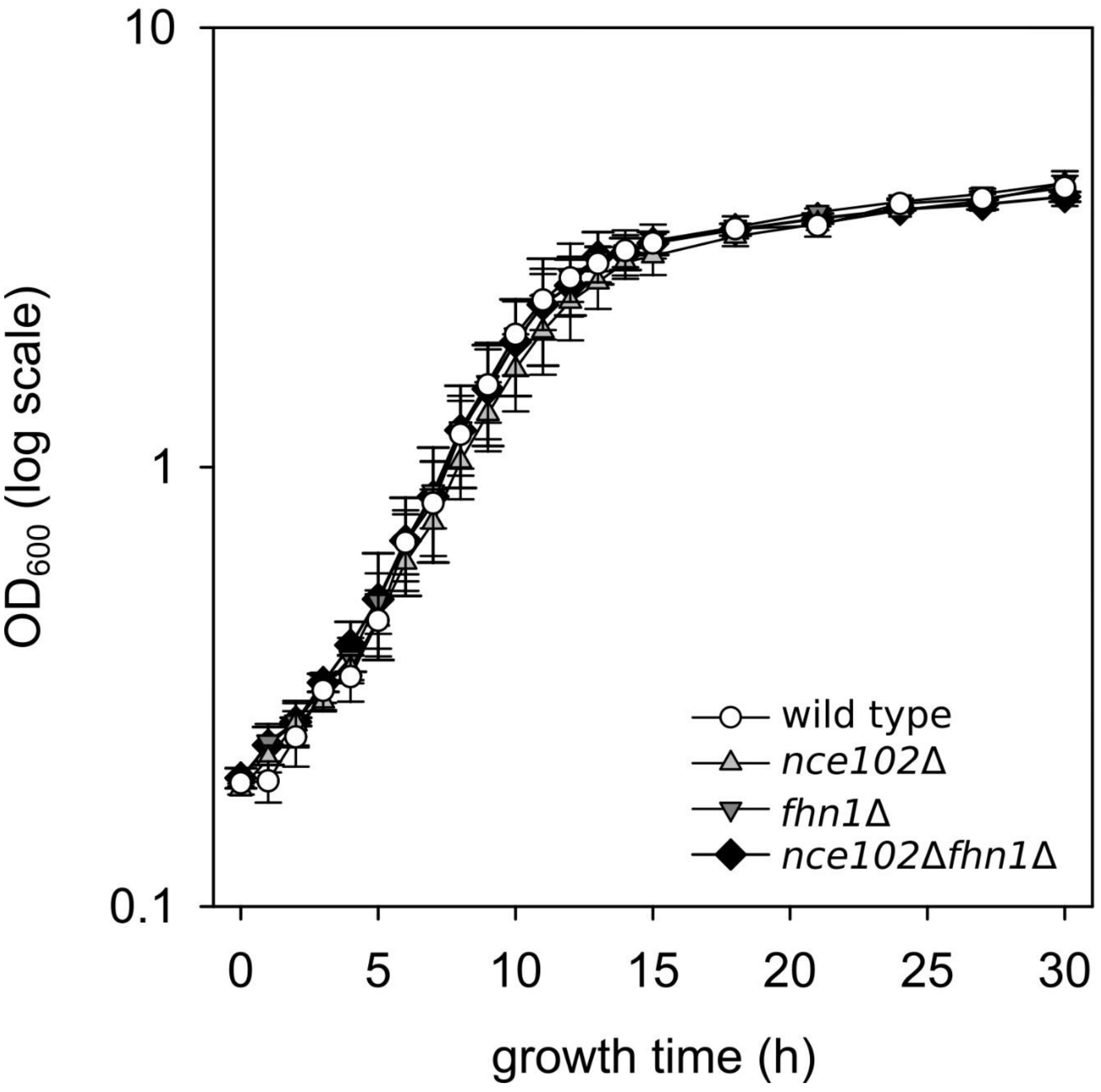

Figure S2. Growth of yeast strains lacking Nce102-like proteins. Growth curves of *nce102*Δ, *fhn1*Δ, and *nce102*Δ*fhn1*Δ strains in a liquid culture were compared to the wild type. All strains exhibited comparable growth rates and reached the diauxic shift at the same time, ~14 h after inoculation. Error bars indicate standard deviations from three biological replicates.
